# The complete genome sequence of the rumen methanogen *Methanobrevibacter millerae* SM9

**DOI:** 10.1186/s40793-016-0171-9

**Published:** 2016-08-17

**Authors:** William J. Kelly, Diana M. Pacheco, Dong Li, Graeme T. Attwood, Eric Altermann, Sinead C. Leahy

**Affiliations:** Rumen Microbiology, Animal Science, AgResearch Limited, Tennent Drive, Private Bag 11008, Palmerston North, 4442 New Zealand

**Keywords:** Methanogen, Methane, Rumen, *Methanobrevibacter millerae*

## Abstract

*Methanobrevibacter millerae* SM9 was isolated from the rumen of a sheep maintained on a fresh forage diet, and its genome has been sequenced to provide information on the phylogenetic diversity of rumen methanogens with a view to developing technologies for methane mitigation. It is the first rumen isolate from the *Methanobrevibacter gottschalkii* clade to have its genome sequence completed. The 2.54 Mb SM9 chromosome has an average G + C content of 31.8 %, encodes 2269 protein-coding genes, and harbors a single prophage. The overall gene content is comparable to that of *Methanobrevibacter ruminantium* M1 and the type strain of *M. millerae* (ZA-10^T^) suggesting that the basic metabolism of these two hydrogenotrophic rumen methanogen species is similar. However, *M. millerae* has a larger complement of genes involved in methanogenesis including genes for methyl coenzyme M reductase II (*mrt*AGDB) which are not found in M1. Unusual features of the *M. millerae* genomes include the presence of a tannase gene which shows high sequence similarity with the tannase from *Lactobacillus plantarum*, and large non-ribosomal peptide synthase genes. The *M. millerae* sequences indicate that methane mitigation strategies based on the *M. ruminantium* M1 genome sequence are also likely to be applicable to members of the *M. gottschalkii* clade.

**Electronic supplementary material:**

The online version of this article (doi:10.1186/s40793-016-0171-9) contains supplementary material, which is available to authorized users.

## Introduction

Ruminant livestock such as cattle and sheep produce methane as a product of enteric fermentation and ruminant-derived methane accounts for almost 30 % of New Zealand’s anthropogenic greenhouse gas emissions. Methane is produced by methanogenic archaea, and sequencing of 16S rRNA gene amplicons has shown that members of the orders *Methanobacteriales* and *Methanomassiliicoccales* are the dominant methanogens in the rumens of farmed New Zealand ruminants [1, 2]. Among the *Methanobacteriales* two different *Methanobrevibacter* species (or clades of very closely related species) constitute the bulk of the population. These two clades are the *Methanobrevibacter gottschalkii* clade (*M. gottschalkii*, *M. millerae* and *M. thaueri*) and the *Methanobrevibacter ruminantium* clade (*M. olleyae* and *M. ruminantium*) with a mean abundance of 42.4 and 32.9 % respectively [2]. These *Methanobrevibacter* species produce methane hydrogenotrophically using hydrogen or formate formed during the fermentation of ingested feed by other members of the rumen microbiota [1]. To mitigate emissions of methane from ruminants into the atmosphere, strategies are being developed to reduce the number or activity of methanogens in the rumen. These mitigation strategies include the development of vaccines and inhibitors based on genome sequences of key methanogens [3]. We have previously used the genome sequence of the type strain of *M. ruminantium* to identify methane mitigation targets [4] and here we present the genome sequence of *M. millerae* SM9, a rumen representative of the *M. gottschalkii* clade.

### Organism information

#### Classification and features

**Fig. 1:**
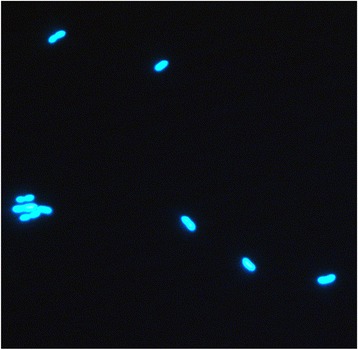
Morphology of *M. millerae* SM9. Micrograph of *M. millerae* SM9 cells captured at 100× magnification using UV illumination to show F420 fluorescence

**Fig. 2:**
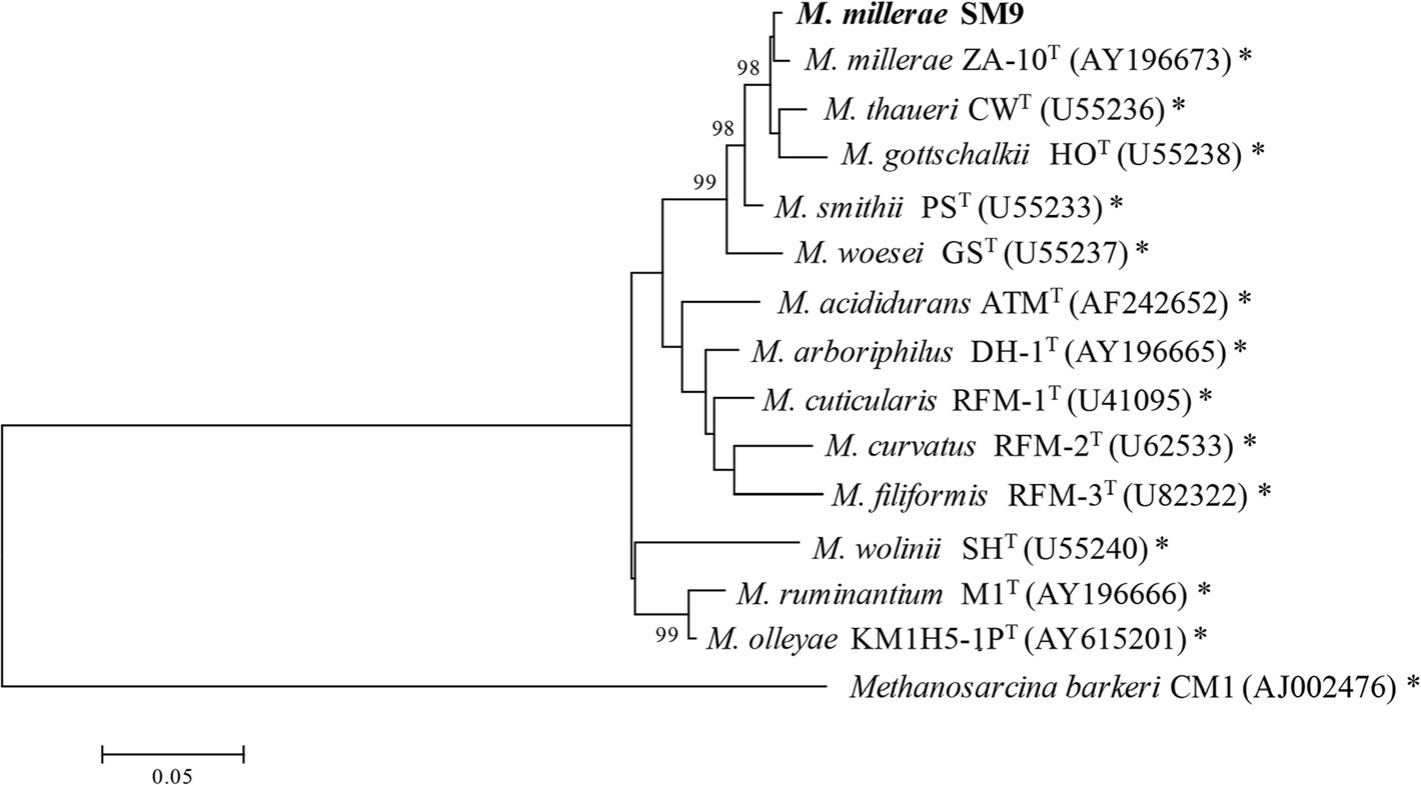
Phylogenetic tree highlighting the position of *M. millerae* SM9 relative to the type strains of the other species within the genus *Methanobrevibacter.* The evolutionary history was inferred by using the Maximum Likelihood method based on the General Time Reversible model [38]. The tree with the highest log likelihood (−4507.7026) is shown. The percentage of trees in which the associated taxa clustered together is shown next to the branches. Initial tree(s) for the heuristic search were obtained automatically by applying Neighbor-Join and BioNJ algorithms to a matrix of pairwise distances estimated using the Maximum Composite Likelihood (MCL) approach, and then selecting the topology with superior log likelihood value. A discrete Gamma distribution was used to model evolutionary rate differences among sites [5 categories (+G, parameter = 0.2484)]. The tree is drawn to scale, with branch lengths measured in the number of substitutions per site. The analysis involved 15 nucleotide sequences. All positions with less than 95 % site coverage were eliminated. That is, fewer than 5 % alignment gaps, missing data, and ambiguous bases were allowed at any position. There were a total of 1206 positions in the final dataset. Evolutionary analyses were conducted in MEGA6 [39]. Species with strain genome sequencing projects registered in the Genomes Online Database (GOLD) [40] are labeled with an asterisk

*Methanobrevibacter millerae* SM9 was isolated from the rumen of a sheep maintained on a fresh forage diet [5]. SM9 cells are Gram positive, non-motile coccobacilli occurring singly or in pairs (Fig. 1). Although originally described as *Methanobrevibacter* sp. [5] or *M. smithii* [6], the 16S rRNA from SM9 is 99 % similar to the *M. millerae* type strain ZA-10^T^ (DSM 16643) [7] and as such SM9 can be considered as a strain of *M. millerae* (Fig. 2). Additional characteristics of *M. millerae* SM9 are shown in Table 1.

**Table 1:** Classification and general features of *Methanbtevibacter millerae* SM9 [41]

MIGS ID	Property	Term	Evidence code^a^
	Classification	Domain: Archaea	TAS [42]
		Phylum: *Euryarchaeota*	TAS [43]
		Class: *Methanobacteria*	TAS [44]
		Order: *Methanobacteriales*	TAS [45, 46]
		Family: *Methanobacteriaceae*	TAS [45]
		Genus: *Methanobrevibacter*	TAS [45]
		Species: *Methanobrevibacter millerae*	TAS [7]
		strain: SM9	
	Gram stain	Positive	TAS [7]
	Cell shape	Coccobacilli	IDA
	Motility	Non-motile	NAS
	Sporulation	Not reported	IDA
	Temperature range	36–42 °C	NAS
	Optimum temperature	38 °C	NAS
	pH range; Optimum	7.0–8.0; 6.8	NAS
	Carbon source	CO_2_, Acetate	IDA
MIGS-6	Habitat	Sheep rumen	TAS [5]
MIGS-6.3	Salinity	Not reported	
MIGS-22	Oxygen requirement	Anaerobic	IDA
MIGS-15	Biotic relationship	Symbiont	TAS [5]
MIGS-14	Pathogenicity	Non-pathogen	NAS
MIGS-4	Geographic location	Palmerston North, New Zealand	IDA
MIGS-5	Sample collection	Not reported	
MIGS-4.1	Latitude	−40.35 (40°21'00"S)	IDA
MIGS-4.2	Longitude	+175.61 (175°36'36"E)	IDA
MIGS-4.4	Altitude	30 M	IDA

^a^Evidence codes - IDA: Inferred from Direct Assay; TAS: Traceable Author Statement (i.e., a direct report exists in the literature); NAS: Non-traceable Author Statement (i.e., not directly observed for the living, isolated sample, but based on a generally accepted property for the species, or anecdotal evidence). These evidence codes are from the Gene Ontology project [47]

## Genome sequencing information

### Genome project history

*Methanobrevibacter millerae* SM9 was selected for genome sequencing on the basis of its phylogenetic position relative to other methanogens belonging to the family *Methanobacteriaceae*, and falls within the *M. gottschalkii* clade of rumen methanogens. The genome sequence of SM9 is being used to underpin the development of technologies to mitigate methane emissions from ruminant livestock. A summary of the genome project information is shown in Table 2 and Additional file 1: Table S1. The 2.73 Mb draft genome sequence of *M. millerae* ZA-10^T^ (JGI IMG/ER genome ID 2593339167) was produced by the Hungate1000 project [8] and used for comparison with SM9.

**Table 2:** Project information

MIGS ID	Property	Term
MIGS-31	Finishing quality	High-quality, closed genome
MIGS-28	Libraries used	Paired-end and mate pair libraries
MIGS-29	Sequencing platforms	454 GS FLX Titanium chemistry
MIGS-31.2	Fold coverage	213×
MIGS-30	Assemblers	Newbler
MIGS-32	Gene calling method	Glimmer and BLASTX
	Locus Tag	sm9
	Genbank ID	CP011266
	Genbank Date of Release	22^nd^ December 2015
	GOLD ID	Gp0007703
	BIOPROJECT	PRJNA49589
MIGS 13	Source Material Identifier	*Methanobrevibacter millerae* SM9
	Project relevance	Ruminant methane emissions

### Growth conditions and genomic DNA preparation

SM9 was grown in BY medium [9] with added SL10 Trace Elements solution (1 ml l^−1^) [10], selenite/tungstate solution (final concentrations of selenite and tungstate were 3 and 4 μg l^−1^ respectively) [11] and Vitamin 10 solution (0.1 ml added to 10 ml culture before inoculation) [4]. Hydrogen was supplied as the energy source by pumping the culture vessels to 180 kPa over pressure with an 80:20 mixture of H_2_:CO_2_. Genomic DNA was extracted from freshly grown cells using a modified version of a liquid N_2_ freezing and grinding method as described previously [12], and purified using the Qiagen Genomic-Tip 500 Maxi kit (Qiagen, Hilden, Germany). Genomic DNA was precipitated by the addition of 0.7 vol isopropanol, and collected by centrifugation at 12,000 × *g* for 10 min at room temperature. The supernatant was removed, and the DNA pellet was washed in 70 % ethanol, re-dissolved in TE buffer (10 mM Tris–HCl, 1 mM EDTA pH 7.5) and stored at −20 °C until required.

### Genome sequencing and assembly

The complete genome sequence of SM9 was determined using pyrosequencing of a paired-end 454 GS-FLX sequence library and a mate-pair 454 GS FLX with Titanium chemistry sequence library (Macrogen, Korea). Pyrosequencing reads provided 213× coverage of the genome and were assembled using the Newbler assembler version 2.0 (Roche 454 Life Sciences, USA). The assembly process resulted in 52 contigs across 1 scaffold. Gap closure was managed using the Staden package [13] and gaps were closed using additional Sanger sequencing by standard and inverse PCR based techniques. A total of 169 additional reactions were used to close gaps and to improve the quality of the genome sequence to ensure correct assembly and to resolve any remaining base-conflicts. Assembly validation was confirmed by pulsed-field gel electrophoresis (data not shown) as described previously [14], using the enzyme MluI which cuts the SM9 chromosome at 16 sites.

### Genome annotation

A GAMOLA/ARTEMIS [15, 16] software suite was used to manage genome annotation. Protein-encoding open reading frames were identified using the ORF-prediction program Glimmer [17] and BLASTX [18, 19]. A manual inspection was performed to verify or, if necessary, redefine the start and stop codons of each ORF. Assignment of protein function to ORFs was performed manually using results from the following sources; BLASTP [18] to both a non-redundant protein database provided by the National Centre for Biotechnology Information [20] and Clusters of Orthologous Groups database [21]. HMMER [22] was used to identify protein motifs to both the PFAM [23] and TIGRFAM [24] libraries. TMHMM [25], (http://www.cbs.dtu.dk/services/TMHMM/) was used to predict transmembrane sequences, and SignalP, version 4.1 [26] was used for the prediction of signal peptides. Ribosomal RNA genes were detected on the basis of BLASTN searches to a custom GAMOLA ribosomal database. Transfer RNA genes were identified using tRNAscan-SE [27]. The genome sequence was prepared for NCBI submission using Sequin [28], and the adenine residue of the start codon of the Cdc6-1 replication initiation protein (sm9_0001) gene was chosen as the first base for the genome. Synteny plots were generated using the program MUMmer, version 3.07 [29]. Only scaffold sequence information greater than 50 kb from the draft genome of *M. millerae* ZA-10^T^ (JGI IMG/ER genome ID 2593339167) was used in the syntheny analysis. The number of shared and unique genes between SM9 and ZA-10^T^ was calculated based on OrthoMCL analysis [30].

## Genome properties

**Fig. 3:**
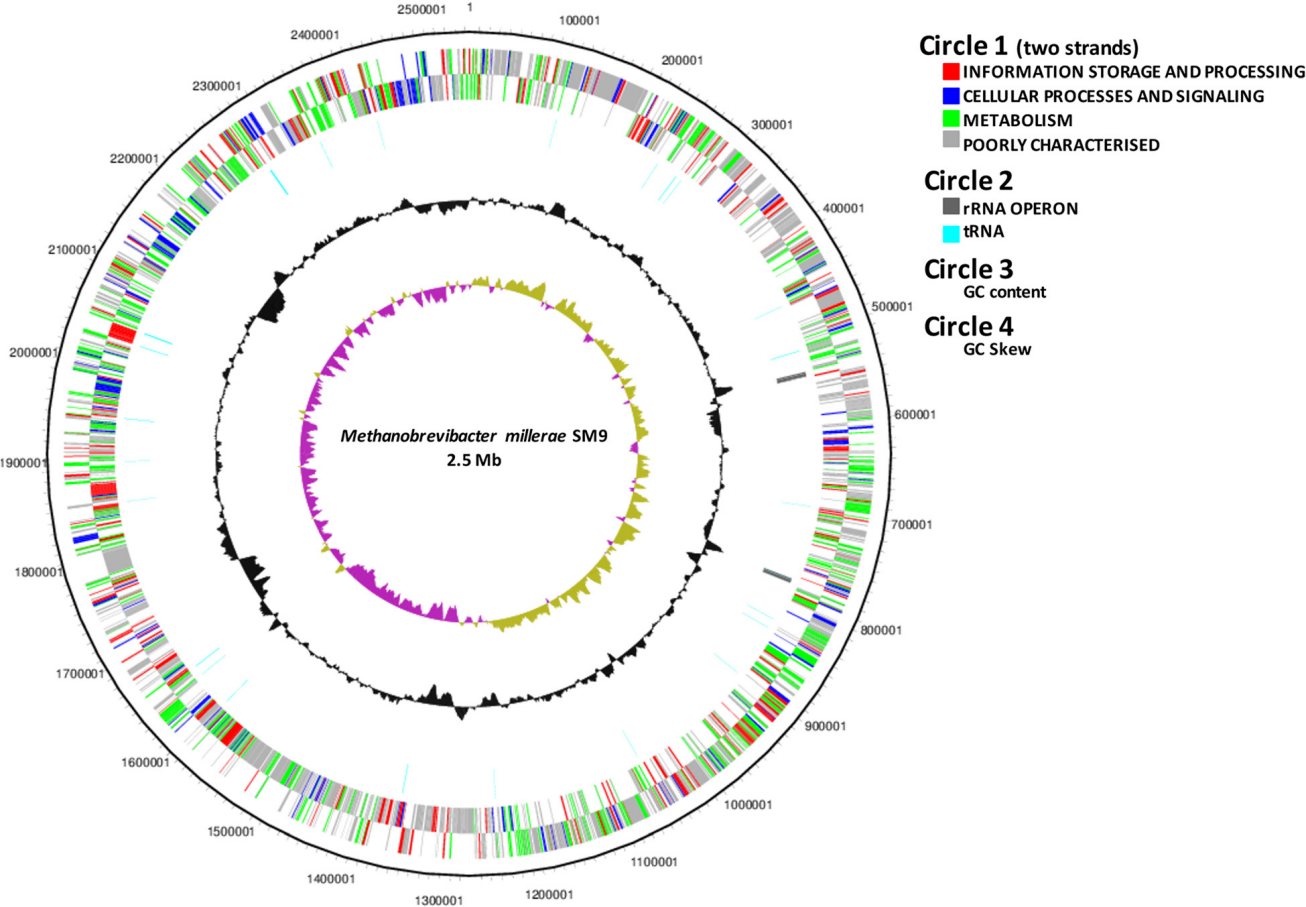
Genome atlas for *M. millerae* SM9. The circles from the outside represent: (1) forward and reverse coding domain sequences, the colour coding of the CDS represent different Clusters of Orthologous Groups categories; (2) rRNA and tRNA; (3) % GC plot; (4) GC skew [(GC)/(G + C)]

The genome of *M. millerae* SM9 consists of a single 2,543,538 base pair (bp) circular chromosome with an average G + C content of 31.8 %. A total of 2370 genes were predicted, of which 2269 were protein-coding genes. The properties and statistics of the SM9 genome are summarized in Tables 3 and 4, and the nucleotide sequence has been deposited in GenBank under accession number CP011266. The SM9 genome contains an integrated 49 kb prophage (sm9_0421-sm9_0483). Most of the genes in this region are predicted to encode hypothetical proteins together with an integrase, a MCM family protein, a terminase, restriction-modification system components and a predicted endoisopeptidase that may function as a lytic enzyme (sm9_0468). There is no homology between this prophage region and that found in the genome of *Methanobrevibacter ruminantium* M1 [4]. The genome atlas for *M. millerae* SM9 is shown in Fig. 3.

**Table 3:** Genome statistics

Attribute	Value	% of Total
Genome size (bp)	2,543,538	100.00
DNA coding (bp)	2,225,085	87.48
DNA G + C (bp)	809,122	31.81
DNA scaffolds	1	100.00
Total genes	2,370	100.00
Protein coding genes	2,269	95.73
RNA genes	47	1.98
Pseudo genes	54	2.28
Genes with function prediction	1,568	66.16
Genes assigned to COGs	1,470	64.79
Genes with Pfam domains	1,951	85.99
Genes with signal peptides	135	5.95
Genes with transmembrane helices	544	23.98
CRISPR repeats	2

**Table 4:** Number of genes associated with the general COG functional categories

Code	Value	% of total^a^	Description
J	145	6.39	Translation
A	0	0.00	RNA processing and modification
K	88	3.88	Transcription
L	129	5.69	Replication, recombination and repair
B	3	0.13	Chromatin structure and dynamics
D	6	0.26	Cell cycle control, mitosis and meiosis
V	37	1.63	Defense mechanisms
T	15	0.66	Signal transduction mechanisms
M	67	2.95	Cell wall/membrane biogenesis
N	4	0.18	Cell motility
U	9	0.40	Intracellular trafficking and secretion
O	45	1.98	Posttranslational modification, protein turnover, chaperones
C	162	7.14	Energy production and conversion
G	48	2.12	Carbohydrate transport and metabolism
E	114	5.02	Amino acid transport and metabolism
F	46	2.03	Nucleotide transport and metabolism
H	90	3.97	Coenzyme transport and metabolism
I	28	1.23	Lipid transport and metabolism
P	59	2.60	Inorganic ion transport and metabolism
Q	25	1.10	Secondary metabolites biosynthesis, transport and catabolism
R	205	9.03	General function prediction only
S	145	6.39	Function unknown
-	800	35.21	Not in COGs

^a^The total is based on the total number of protein coding genes in the genome

## Insights from the genome sequence

**Fig. 4:**
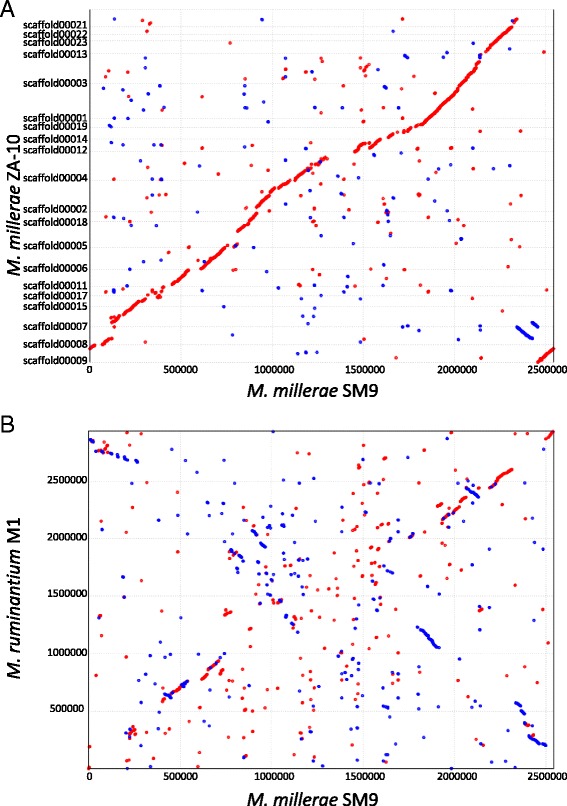
Synteny analysis. Alignment of the *M. millerae* SM9 genome against the draft genome of *M. millerae* ZA-10^T^ (**a**) and the complete genome of *M ruminantium* M1 (**b**). Whenever the two sequences agree, a coloured line or dot is plotted. If the two sequences were perfectly identical, a single line would go from the bottom left to the top right. Units displayed in base-pairs

The genome of *M. millerae* SM9 shows a high level of synteny (Fig. 4a) with that of *M. millerae* ZA-10^T^. Comparison of the ORFeome of SM9 with that of ZA-10 shows a core genome of 1783 genes with 486 unique genes in SM9 and 600 in ZA-10.

Although the genomes of *M. millerae* SM9 and *M. ruminantium* M1 do not show significant synteny (Fig. 4b), their gene contents are comparable suggesting that the basic metabolism of these two hydrogenotrophic rumen methanogen species is similar. However, there are important differences between the methanogenesis genes from the two species. *M. millerae* SM9 has the same set of methanogenesis genes as *M. ruminantium* M1, but also has several genes not found in M1 including an additional gene cluster containing the methyl coenzyme M reductase II (*mrt*AGDB) genes together with a second copy of F420-dependent methylenetetrahydromethanopterin dehydrogenase (*mtd*), and a second set of formate dehydrogenase genes (*flp*ABD). Compared to M1, SM9 also has additional copies of the methanogenesis genes *hmd*, *hdr*ABC and *mtr*H and the methanogenesis marker proteins 5 and 8. The two *M. millerae* strains have the same complement of methanogenesis genes but the *mrt*AGDB-*mtd* and *flp*ABD genes are not co-located in strain ZA-10. It is possible that the difference in methanogenesis genes may allow *M. ruminantium* and *M. millerae* to occupy different niches within the rumen environment [4], and explain why both groups are always found in ecological studies of rumen methanogens [31]. Genome sequences from further strains belonging to the *M. gottschalkii* and *M. ruminantium* clades are required to determine if these differences are common features of the two groups.

The biosynthetic genes for most cofactors are conserved between the SM9 and M1 strains with the exceptions being biotin, cobalamin and coenzyme M. M1 encoded genes for biotin biosynthesis of bacterial origin [4], but these are not present in SM9 or ZA-10, although both *M. millerae* strains contain a BioY transporter believed to be responsible for biotin uptake. Many of the cobalamin biosynthesis genes in M1 were clustered and of bacterial origin, whereas SM9 and ZA-10 also have a full complement of cobalamin biosynthesis genes but their organization is different and they are spread throughout the genome. M1 is unable to synthesise coenzyme M because it lacks key genes, but SM9 and ZA-10 have the five genes necessary (*com*A, *com*B, *com*C, *com*D and *com*E) for coenzyme M synthesis.

The pseudomurein biosynthesis genes found in SM9 and ZA-10 are similar to those reported for M1, and their genomes also encode genes for the production of several different cell wall associated polysaccharides. Unique genes in strain ZA-10 include a cluster of four genes that have no methanogen matches. These are IE19DRAFT_01711-4 and include genes encoding phosphoenolpyruvate mutase and phosphonopyruvate decarboxylase whose location suggests they could be involved in modification of cell wall polysaccharides. Both strains contain numerous adhesin-like proteins but while the the role of these is not known it seems likely that they are important for methanogen ecology in the rumen [32]. Many of these proteins are very large (sm9_1600 is predicted to encode a protein of 7805 amino acid residues) and their production likely represents a considerable metabolic burden on the cell.

Tannins are polyphenolic secondary metabolites found in a variety of plants used as forages for ruminants, and are known to have significant effects on animal nutrition [33]. One of these effects is to reduce methane production [34] and tannins have been shown to have direct inhibitory effects on methanogens belonging to the genus *Methanobrevibacter* [35]. Some microorganisms are resistant to tannins and encode the enzyme tannin acyl hydrolase (tannase) which catalyses the hydrolysis of the galloyl ester bond of tannins. The best studied bacterial tannases are those from *Lactobacillus plantarum* which have been biochemically and structurally characterized [36, 37], but tannases have not been reported from methanogens. Both *M. millerae* genomes contain genes (sm9_1028 and IE19DRAFT_01487) predicted to encode signal peptide-containing proteins with high sequence identity (50 %) to TanA_Lp_ from *L. plantarum*. These proteins contains the conserved sequence motifs involved in catalysis and substrate-binding that have been identified in TanA_Lp_ [36]. We hypothesize that strains of *M. millerae* have the ability to produce an extracellular tannase which enables them to tolerate tannins encountered in the rumen, and that this protein has been acquired by horizontal gene transfer from another member of the rumen microbial community. A Blast search of the predicted tannase from SM9 also shows homology with predicted proteins from a number of rumen bacteria sequenced in the Hungate1000 project [8] including organisms belonging to the phyla *Actinobacteria* (*Corynebacterium* and *Slackia* sp.) and *Firmicutes* (*Butyrivibrio*, *Oribacterium*, *Pseudobutyrivibrio* and *Streptococcus*). In all cases the residues important for activity are conserved.

The SM9 genome has two non-ribosomal peptide synthase genes (sm9_0755 and _0760 predicted to encode proteins of 2605 and 2394 amino acids) located close together, convergently transcribed, separated by transporters and bounded by transposases. The predicted protein from sm9_0755 is similar (81 % amino acid identity) to the one predicted to be encoded by mru_0068 from *M. ruminantium* M1 [4]. In contrast the ZA-10 genome has three non-ribosomal peptide synthase genes (IE19DRAFT_00420, _00763 and _01910 predicted to encode proteins of 4187, 4390 and 2573 amino acids) that differ from those found in SM9. The predicted protein from IE19DRAFT_00420 is a close match (89 % amino acid identity) to the one predicted to be encoded by mru_0351 from M1 [4].

## Conclusions

The species *M. millerae* belongs to the *Methanobrevibacter gottschalkii* clade of rumen methanogens and the availability of genome sequences for strains SM9 and ZA-10 provide valuable information for developing methane mitigation strategies targeting this group. While the *M. millerae* genome is largely similar to that of *M. ruminantium* M1 it is notable that strains SM9 and ZA-10 have a larger complement of methanogenesis genes. The *M. gottschalkii* and *M. ruminantium* clades are the dominant hydrogenotrophic methanogens in the rumen and these differences in methanogenesis genes may allow them to occupy different niches in the rumen environment. Genome sequences from additional rumen strains will establish if the observations based on these representatives are characteristic of the two clades. Both *M. millerae* genomes contain a tannase of bacterial origin which may represent an adaptation to the rumen environment as tannin containing plants are an important component of fresh forages, and tannins are known to have an inhibitory effect on methanogens. The overall similarity between the genomes of *M. millerae* and *M. ruminantium* M1 suggests that the strategies based on the M1 genome should be generally applicable to methanogens belonging to the *M. gottschalkii* clade.

## Additional file

Additional file 1: Table S1. Associated MIGS record for M. millerae SM9, which links to the SIGS supplementary content website. (DOC 70 kb)

## Abbreviations

H_2_: Hydrogen
CO_2_: Carbon dioxide
N_2_: Nitrogen
TE: Tris Ethylenediaminetetraacetic acid
